# Bath and Her Waters

**Published:** 1889-03-16

**Authors:** 


### BATH AND HER WATERS.

Mr. S. Craddock, M.R.C.S., has published a paper in pamphlet form, read before the Bath and Bristol branch of the British Medical Association, entitled "Bath and her Thermal Waters." The author's object is to endeavour to define, categorically, the real therapeutical value of the Bath waters. On perusing some of the books that have been written on these waters, the reader cannot but be surprised at the multitude of diseases for which they are reputed to be serviceable. But Mr. Craddock regards the Bath waters as suitable for particular cases, and not adapted for universal application; and to this it may be added, as not adapted for universal application in any particular malady. Perhaps the disease in which the Bath waters are most useful is gout, whether regular or irregular in its manifestations. Lead poisoning, such as occurs to plumbers, painters, and farm labourers who drink cider contaminated with lead, is also always benefited. So is chronic muscular rheumatism, and rheumatoid arthritis. But as regards the latter disease, great care should be taken in the selection of cases, whilst those of a decided neurotic origin should be positively excluded. Anæmia, or want of good red blood, frequently affecting females, is one of the maladies for which the Bath waters have long been more or less praised; but the author does not join in this estimate, regarding the Bath climate calculated to undo the beneficial effects which may result from the waters. The climate of Bath is comparatively warm, moist, and somewhat changeable. There is, especially in the lower parts, an excess of humidity in summer and autumn as compared with places eastward and westward, but a deficiency in the winter and spring. The average rainfall is 30 inches annually, of which nearly six inches fall in three spring months, nearly seven in the summer, about eight in the autumn, and nine in the winter. The prevailing winds are from the west and north-west, in which directions the city is well protected. Yet when blowing hard the direct east wind will rush down the sheltering but distant slope of Claverton, passing over Widcomb, to envelop and annoy Bathwick first, and then the older city. The frequency of such wind is an unfortunate meteorological feature of Bath. The mean temperature of spring is 49 deg., of summer 61 deg., of autumn 50 deg., and of winter 46 deg. In several of the colder months the comparatively high temperature evidences the mildness of the climate. Mr. Craddock does not tell us all this, which has been taken from another source. Mr. Craddock unhesitatingly says that Bath is relaxing, especially during the months of June, July, and August. The humidity which arises from the River Avon he considers a barrier to the successful treatment of chest cases, particularly phthisis. He admits, however, that a divergence of opinion exists with regard to struma, one writer extolling the Bath waters in its treatment, whilst another considers their use inadvisable. Other facts are mentioned as showing that complete harmony does not exist among medical advisers as to the use of the Bath waters.

It is refreshing to find a practitioner at a health resort stating what diseases are not benefited as well as what are. As a rule the tendency of writers on health resorts is to extol their particular climate, or waters, without much limitation. But, as a matter of fact, there is no place which will suit all invalids, or even which will suit all invalids suffering from a similar malady. Individuality and idiosyncrasy must be consulted. It should be considered whether a sheltered or an open spot is required, whether the air should be bracing or mild and relaxing. Also whether exercise can be taken under suitable circumstances. The nature of the soil demands consideration, as dampness depends more on the ground stratum and on drainage than the actual amount of rain. All these and many other considerations are required before an invalid visits a health resort. It is little use locating in a bad climate for the sake of particular waters. In or near Bath, however, a person may find a variety of climates, although this entails horses, carriages, servants, and a style of living which all cannot afford. Landor described Bath as "the only place worth living in except Florence." Mr. Craddock, a medical resident at Bath, does not endorse this estimate. He honestly tells us what the locality is good for, and what it is not good for, and the possession of his pamphlet would doubtless prevent disappointment to many, while confirming the hesitating as to their choice or otherwise of a sojourn in the city where Romans bathed, and which, as Macaulay observed, "charms every eye."

